# Cottage Hospitals

**Published:** 1887-11-26

**Authors:** 


### COTTAGE HOSPITALS.

Miss Nicholson writes to complain that: "Burdett's book on Cottage Hospitals (J. and A. Churchill) is very vague in many matters about setting a hospital going, and I hope to begin one before Christmas, so naturally wish to have the best rules." She asks the following questions: "Will you give me some information about management and rules for cottage hospitals? Burdett's "Cottage Hospitals" does not enter into minute particulars as to advantages of lady or working matrons; also rules and expense of starting one in quite remote country village. As I am interested in one to be soon started, any particulars as to management most acceptable."

*** We have referred to "Cottage Hospitals," and find that it contains all the information here asked for in considerable detail. We refer our correspondent as to (1) "Setting a hospital going," to pages 10, 11, and 361; (2) "Management and rules," to pages 38, 197 *et seq.*, 444 *et seq.*; (3) "Lady or working matron," to pages 183 *et seq.*, 367 and 326; (4) "Expense of starting one," to pages 361 and 496 *et seq.* Nothing could be fuller than the information here given. Will some of our cottage hospital friends send Miss Nicholson, Putney Heath, S.W., one of their last reports and a copy of the rules.

